# EBM BLS: Self-collected Human Papillomavirus Cervical Cancer Screening Is Non-Inferior to Clinician-Collected Samples

**DOI:** 10.1007/s11606-025-09830-x

**Published:** 2025-10-07

**Authors:** Claire Ruben, Sweta Narasimhan, Eric Nolan

**Affiliations:** 1 College of Medicine, The Ohio State University Wexner Medical Center, Columbus, OH USA; 2 Baylor Scott and White Medical Center, Waxahachie, TX USA; 3 Division of Hospital Medicine, Department of Internal Medicine, College of Medicine, The Ohio State University Wexner Medical Center, Columbus, OH USA

**Keywords:** Cancer prevention, Cervical cancer screening, HPV testing, Self-collection

**Source Article:** Polman NJ, et al. Performance of human papillomavirus testing on self-collected versus clinician-collected samples for the detection of cervical intraepithelial neoplasia of grade 2 or worse: a randomised, paired screen-positive, non-inferiority trial. Lancet Oncol. 2019 Feb;20(2):229–238. https://doi.org/10.1016/S1470-2045(18)30,763–0. PMID: 30,658,933.

## Why This is Important

- In the U.S., 28% of women are not up to date on cervical cancer screening.^1^ Optimal screening could reduce cervical cancer mortality by up to 97%.^2^
- Nearly all cervical cancer results from human papillomavirus (HPV).^1^ Cervical intraepithelial neoplasia (CIN), classified as low grade (CIN-1) and high-grade (CIN-2 or 3), precedes cancer and is detected via colposcopy.
- The FDA-approved office-based HPV self-collection in May 2024. United States Preventive Services Task Force (USPSTF) draft guidelines include HPV self-collection every 5 years for females aged 30 to 65.^1^
- Cervical cancer screening is traditionally office-based using HPV testing or cytology. Self-collected HPV screening may increase uptake by reducing access barriers.
- The IMPROVE study, a randomized, paired, screen-positive, noninferiority trial, compared self-collected HPV screening to clinician-screening.^3^ It was the only trial in the USPSTF review that evaluated all three key outcomes of self-collected HPV testing: accuracy, potential harms, and adherence to follow-up care.^1^

## Intervention

- Participants were randomized 1:1 and stratified by age. The self-collection group received a brush-based kit and returned samples by mail. The clinician-collection group underwent office-based HPV testing and cytology.
- A screen-positive, cross-testing design was used: HPV-positive participants in the self-collection group had clinician collected HPV and cytology. HPV-positive clinician collection participants had reflex cytology on samples and provided self-collected HPV testing.

## Results

**Figure 1:**
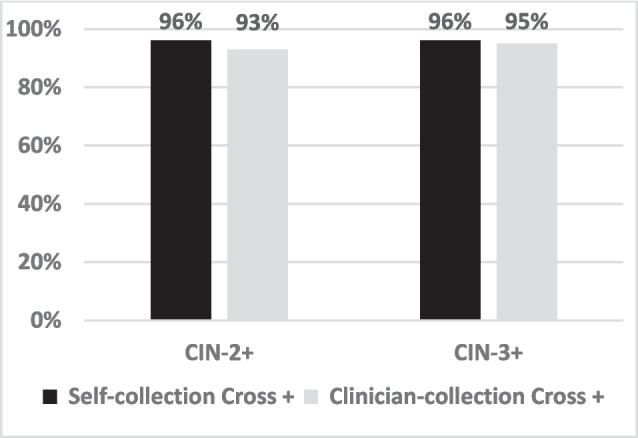
Accuracy of self- and clinician-collected co-testing. Percentage of participants with CIN-2 + and CIN-3 + who were originally HPV-positive via self- (black) and clinician-collection (grey) and subsequently tested positive with the other method

- 16,410 females of 187,473 screened opted-in and were randomized.
- 93% (7643 of 8193) in the self-collection group and 77% (6282 of 8168) in the clinician-collection group provided a HPV sample.
- HPV was detected in 7.4% self-collected samples and 7.2% clinician-collected samples, with similar prevalence across age cohorts.
- Detection rates of CIN-2 or worse (CIN-2 +) were similar between self-collection (1.5%) and clinician-collection (1.5%, RR 0.99 [95% CI 0.75–1.31]). Similar findings were observed for detection of CIN-3 or worse (CIN-3 +).
- HPV-positive cross-testing showed comparable results between self-sampling and clinician sampling. Among participants with CIN-2 +, 96% of HPV-positive self-sampling and 93% of clinician-sampling participants were subsequently HPV-positive with the other method. For CIN-3 +, 96% of self-collection and 95% of clinician collection participants were cross positive (Fig. 1).
- The relative sensitivity and specificity of self-collected HPV testing were similar to clinician-collection for detecting CIN-2 + (sensitivity 0.96, specificity 1.00) and CIN-3 + (0.99, 1.00).

## Study Design

### Setting

- Included females aged 29–61 undergoing routine cervical cancer screening through the national cervical cancer screening program in multiple regions of the Netherlands.

### Exclusion Criteria

- Previous hysterectomy, childbirth less than 6 months prior, and current pregnancy.

### Methods

- Participants, physicians, and researchers knew group assignments.
- HPV polymerase chain reaction testing evaluated for 14 high-risk HPV strains.
- Positive HPV testing prompted cytology. If cytology was abnormal, colposcopy was performed; those with normal colposcopy required repeat 6-month cytology.
- Primary outcomes were the detection of CIN-2 or higher-grade histology or cancer (CIN-2 +), and CIN-3 or cancer (CIN-3 +).

## Study Quality and Application to Patients

- The USPSTF rating of this trial is good.
- Strengths include randomization with age stratification and screen positive cross-testing prior to colposcopy.
- Limitations include a low participation rate among invited women, and the study’s setting within the Netherlands’ centralized cervical cancer screening program, which may limit generalizability to less integrated healthcare systems.
- The study used CIN-2 + and CIN-3 + as endpoints. Long-term mortality is the gold standard for assessing screening benefits.
- In this trial, most HPV-positive self-collected participants completed follow-up cytology (98%). Self-collection resulted in higher screening completion and comparable accuracy to clinician-collected samples. These findings align with a growing body of evidence supporting self-collection’s potential to improve screening uptake.^1^
- Most cervical cancer cases occur in unscreened or underscreened individuals. Survivors of intimate partner violence (IPV) comprise 20–30% of females in the US and are disproportionately underscreened. Up to 87% of IPV survivors prefer self-collection, underscoring its potential to improve uptake among vulnerable populations.^4^
- The FDA has approved two office-based HPV self-collection tests. At-home self-collection with mail-in submission may improve screening accessibility.
